# Hyperchloremia, a necessary evil in neurocritical care

**DOI:** 10.1186/s13054-023-04639-8

**Published:** 2023-09-12

**Authors:** Gonzalo Ramírez-Guerrero, Matteo Marcello, Thiago Reis

**Affiliations:** 1 Critical Care Unit, Carlos Van Buren Hospital, San Ignacio #725, Valparaíso, Chile; 2 Nephrology and Dialysis Unit, Carlos Van Buren Hospital, Valparaíso, Chile; 3 Deparment of Medicine, Universidad de Valparaíso, Valparaíso, Chile; 4 International Renal Research Institute of Vicenza (IRRIV Foundation), Department of Nephrology, Dialysis and Kidney Transplantation, San Bortolo Hospital, Vicenza, Italy; 5 Deparment of Nephrology and Kidney Transplantation, Fenix Group, Sao Paulo, Brazil; 6 Laboratory of Molecular Pharmacology, University of Brasília, Brasília, Brazil

Dear Editor:

We read with interest the recent article by Huet et al. A post hoc study from the COBI trial compared the impact of continuous hypertonic (NaCl 20%) saline solution on renal outcomes after traumatic brain injury (TBI), specifically, if a high dose of chloride delivered by hypertonic saline solution was associated with an increased incidence of acute kidney injury. In their conclusion, they question the detrimental effect of chloride on kidney function [1]. However, it is necessary to mention some relevant aspects of acute kidney injury in neurocritical care patients.

1. Hyperchloremia has been hypothesized to cause renal hypoperfusion and AKI by its renal vascular smooth muscle constrictor effect and other mechanisms [1]. Therefore, there is a pathophysiological rationale to support its association with AKI.
  The randomized controlled trials SMART and SALT-ED support this relationship. The participants in the SMART trial who received balanced solutions had 10% lower odds of major adverse kidney events within 30 days. Similar results for SALT-ED, with 18% lower odds of MAKE-30, compared with normal saline. Additionally, those who presented to the emergency department with renal dysfunction or hyperchloremia received the largest benefit from balanced solutions for avoiding AKI. However, these results are limited to a primarily non-neurocritical population. It is important to note that in neurocritical patients the administration of saline solution and not a balanced solution is indicated due to the risk of cerebral edema. However, it is necessary to be attentive to the effects of severe hyperchloremia that neurocritical patients can develop. Nevertheless, Riha et al. [2] demonstrated significantly higher rates of in-hospital mortality in patients with intracerebral hemorrhage who developed moderate hyperchloremia during hypertonic 3% solution treatment. Additionally, the ACETatE trial, a pilot study, decreased the chloride load and the incidence of AKI (11.8% vs 53.3%, *p* = 0.01) using a hypertonic solution with a lower chloride content of 16.4% sodium chloride/sodium acetate vs 23.4% sodium chloride for the treatment of cerebral edema [3]. This study supports the hypothesis that chloride may be a potentiator of AKI in this population.
2. The actual comparison was patients with hyperchloremia vs patients with greater hyperchloremia (e.g., 114.5 ± 6.4 mmol/L vs 122.8 ± 8.1 mmol/L at day 2), making it difficult to find a difference in this scenario.
3. More than 50% of the patients received hypertonic therapy before inclusion, and even both groups received more than 50%, of mannitol, another controversial solution.
4. The definition of AKI only included patients with KDIGO 2 or 3. It is important to mention that the reports of AKI in neurocritical patients show a high incidence of KDIGO 1, which is associated with worse outcomes and worse functional recovery [4]. Failure to report this group may be a factor for not finding differences. When trying to use more severe stages, as in this case, it is possible to classify some AKI cases as negative endpoints because it only achieved KDIGO 1, being really a true AKI case and not just purely functional and transitory. Therefore, defining the AKI outcome in neurocritical patients must be carefully evaluated.
5. Augmented renal clearance is likely to complicate the care of TBI patients with normal plasma creatinine concentrations, limiting our AKI diagnosis by usual markers. Using biomarkers such as NGAL increases our diagnosis of acute tubular injury in patients with normal creatinine after a TBI [5].

Therefore, like the primary study, we believe that the COBI post hoc study should not change our clinical practice by requiring hypertonic solutions in the context of intracranial hypertension. However, we must not think that the increases in chloride will not have repercussions at the kidney level.

## Data Availability

Not applicable.
